# Risk of venous thromboembolism in people admitted to hospital with selected immune-mediated diseases: record-linkage study

**DOI:** 10.1186/1741-7015-9-1

**Published:** 2011-01-10

**Authors:** Sreeram V Ramagopalan, Clare J Wotton, Adam E Handel, David Yeates, Michael J Goldacre

**Affiliations:** 1 Wellcome Trust Centre for Human Genetics, University of Oxford, Oxford, UK; 2 Department of Clinical Neurology, University of Oxford, Oxford, UK; 3 Unit of Health-Care Epidemiology, Department of Public Health, University of Oxford, Oxford, UK

## Abstract

**Background:**

Venous thromboembolism (VTE) is a common complication during and after a hospital admission. Although it is mainly considered a complication of surgery, it often occurs in people who have not undergone surgery, with recent evidence suggesting that immune-mediated diseases may play a role in VTE risk. We, therefore, decided to study the risk of deep vein thrombosis (DVT) and pulmonary embolism (PE) in people admitted to hospital with a range of immune-mediated diseases.

**Methods:**

We analysed databases of linked statistical records of hospital admissions and death certificates for the Oxford Record Linkage Study area (ORLS1:1968 to 1998 and ORLS2:1999 to 2008) and the whole of England (1999 to 2008). Rate ratios for VTE were determined, comparing immune-mediated disease cohorts with comparison cohorts.

**Results:**

Significantly elevated risks of VTE were found, in all three populations studied, in people with a hospital record of admission for autoimmune haemolytic anaemia, chronic active hepatitis, dermatomyositis/polymyositis, type 1 diabetes mellitus, multiple sclerosis, myasthenia gravis, myxoedema, pemphigus/pemphigoid, polyarteritis nodosa, psoriasis, rheumatoid arthritis, Sjogren's syndrome, and systemic lupus erythematosus. Rate ratios were considerably higher for some of these diseases than others: for example, for systemic lupus erythematosus the rate ratios were 3.61 (2.36 to 5.31) in the ORLS1 population, 4.60 (3.19 to 6.43) in ORLS2 and 3.71 (3.43 to 4.02) in the England dataset.

**Conclusions:**

People admitted to hospital with immune-mediated diseases may be at an increased risk of subsequent VTE. Our findings need independent confirmation or refutation; but, if confirmed, there may be a role for thromboprophylaxis in some patients with these diseases.

## Introduction

Venous thromboembolism (VTE), which comprises deep vein thrombosis (DVT) and pulmonary embolism (PE), is thought to account for an estimated 25,000 deaths annually in the UK [1], with a recent study suggesting that the figure might be as high as 60,000 [2]. VTE is a common complication during and after hospitalisation for acute medical illness or surgery [3]. PE accounts for 5 to 10% of deaths in hospitalised patients, making VTE the most common preventable cause of in-hospital death [3]. Risk factors for VTE include immobility, age and obesity [1].

Although VTE has traditionally been considered a surgical condition, the vast majority of hospitalised patients with symptomatic VTE have not undergone recent surgery [4]. Indeed, up to 80% of in-hospital fatal PEs occur in nonsurgical patients [4]. Patients with inflammatory bowel disease [5], rheumatoid arthritis [6], type 1 diabetes [7] and systemic lupus erythematosus (SLE) [8] are at an increased risk of VTE, suggesting that there may be a more general association between immune-mediated diseases and VTE.

To investigate this further, we undertook a record linkage study to determine the risk of VTE in individuals with selected immune-mediated diseases using the long-standing Oxford Record Linkage Study (ORLS) [9] and the more recent English national linked Hospital Episode Statistics (HES) dataset.

## Methods

### Population and data

The Oxford Record Linkage Study (ORLS) [9] includes brief statistical abstracts of records of all hospital admissions (including day cases) in National Health Service (NHS) hospitals, and all deaths regardless of where they occurred, in defined populations within the former Oxford NHS Region. The original ORLS covered the years 1963 to 1998, and is referred to here as ORLS1. The hospital data were collected routinely in the NHS as the region's hospital statistics system and were similar to English national hospital episode statistics (HES). A second dataset, referred to as ORLS2, has been linked and built as the Oxford regional subset of the English national Hospital Episode Statistics (HES), and runs from 1999 to 2008. The data items available for linkage changed between 1998 and 1999 and the two datasets are not themselves linked together. We have also used the complete dataset of the English national HES (1999 to 2008). Death data in each of the three datasets derive from death certificates. The population covered by ORLS1 gradually expanded over time from an initial population of part of Oxfordshire (an approximately 300,000 resident population) to all four counties of the former Oxford NHS region (resident population 2.5 million); and the ORLS2 covers the same four counties. The population of England is 50 million. The datasets used in this study (versions m6v2 for ORLS and v08a for ORLS2 and England) have been constructed by staff in the Oxford Unit of Healthcare-Epidemiology.

The basic methods were the same for the analysis of each disease and are described for rheumatoid arthritis and VTE. A cohort of people with a record of admission or day case care for rheumatoid arthritis was constructed for those with a principal diagnosis of rheumatoid arthritis, as the reason for hospital care, by identifying the first admission, or episode of day case care, for the condition in an NHS hospital during the study period of 1963 to 1998 in ORLS1, 1999 to 2008 in ORLS2, and 1999 to 2008 for the whole of England. The International Classification of Disease (ICD) codes used for each immune-mediated disease can be found in Table 1 and 2. The ICD codes used for VTE (DVT and PE) were: 465 in ICD 7; 450 in ICD8; 415.1, 451.1 and 453.9 in ICD9; I26 and I80.2 in ICD10. A reference cohort was constructed by identifying the first admission for each individual with various other, mainly minor medical and surgical, conditions (listed in Table 2 footnotes). This is based on a 'reference' group of conditions that has been used in other studies of associations between diseases [10-12]. In its design, the standard epidemiological practice was followed, when hospital controls are used, of selecting a diverse range of conditions, rather than relying on a narrow range (in case the latter are themselves atypical in their risk of subsequent disease). As a check, we have studied the risk of VTE in the control conditions within the reference cohort to ensure that the reference cohort does not include control conditions that have atypically high or low VTE rates.

**Table 1:** Number of people in the study with each immune-mediated disease, and percentage of females.

**Exposure cohort (and ICD code^1)^**	**ORLS1**	**ORLS2**	**England**
	
	**Total (% female)**	**Total (% female)**	**Total (% female)**
Addison's disease (255.4)	489 (64)	624 (60)	10,257 (60)
Ankylosing spondylitis (720)	976 (30)	991 (29)	2,2001 (29)
Autoimmune haemolytic anaemia (283)	421 (55)	377 (59)	7,854 (55)
Chronic active hepatitis (571.4)	431 (61)	209 (71)	4512 (70)
Coeliac disease (579.0)	1,722 (61)	2,324 (66)	48,239 (66)
Dermatomyositis, polymyositis (710.3,710.4)	328 (63)	268 (70)	6,002 (63)
Diabetes mellitus under 30 (250)	4,950 (47)	4,410 (48)	79,581 (48)
Goodpasture's syndrome (446.2)	76 (53)	53 (43)	999 (45)
Hashimoto's thyroiditis (245)	524 (87)	384 (90)	8,573 (88)
Idiopathic thrombocytopenia purpura (287.3)	779 (54)	1,221 (57)	25,984 (55)
Multiple sclerosis (340)	3,823 (66)	4,106 (68)	81,950 (68)
Myasthenia gravis (358)	379 (63)	420 (57)	10,024 (51)
Myxoedema (244)	6,738 (82)	23,456 (81)	63,0354 (81)
Pemphigus, pemphigoid (694.4,694.5)	537 (57)	492 (57)	11,480 (57)
Pernicious anaemia (281)	4,131 (63)	1,457 (70)	47,092 (68)
Polyarteritis nodosa (446)	298 (42)	84 (42)	2,088 (43)
Primary biliary cirrhosis (571.6)	359 (77)	367 (83)	9,060 (85)
Psoriasis (696.0,696.1,696.8,696.9)	3,910 (52)	3,365 (48)	85,358 (48)
Rheumatoid arthritis (714)	14,231 (73)	11,241 (72)	268,005 (71)
Scleroderma (710.1)	363 (80)	542 (85)	11,643 (82)
Sjogren's syndrome (710.2)	165 (86)	446 (89)	12,680 (89)
Systemic lupus erythematosus (710.0)	594 (86)	997 (87)	23,544 (87)
Thyrotoxicosis (242)	5,503 (81)	3,986 (89)	91,913 (79)

^1 ^ICD 9 codes for each disease (equivalent codes were used for cases coded in ICD Revisions 7, 8 and 10)

**Table 2:** Rate ratios^1 ^and 95% confidence intervals (CIs) for venous thromboembolism, compared with the control cohort^2^

	Dataset (years)					
**Immune-mediated disease (ICD code^3^)**	**ORLS1 (1963 to 1998) Rate ratio (95% CI) *P*-value**		**ORLS2 (1999 to 2008) Rate ratio (95% CI) *P*-value**		**England (1999 to 2008) Rate ratio (95% CI) *P*-value**	

Addison's disease (255.4)	1.49 (0.79 to 2.55)	0.20	2.96 (1.53 to 5.20)	< 0.001	2.15 (1.86 to 2.47)	< 0.001
Ankylosing spondylitis (720)	1.35 (0.80 to 2.13)	0.26	1.18 (0.56 to 2.17)	0.73	1.93 (1.74 to 2.14)	< 0.001
Autoimmune haemolytic anaemia (283)	2.83 (1.62 to 4.60)	<0.001	3.64 (2.11 to 5.85)	<0.001	3.83 (3.43 to 4.25)	< 0.001
Chronic active hepatitis (571.4)	2.24 (1.25 to 3.70)	0.003	3.99 (1.46 to 8.72)	0.001	2.04 (1.65 to 2.50)	<0.001
Coeliac disease (579.0)	1.35 (0.89 to 1.97)	0.21	1.36 (0.87 to 2.03)	0.17	1.21 (1.11 to 1.33)	<0.001
Dermatomyositis, polymyositis (710.3,710.4)	1.98 (0.99 to 3.55)	0.04	2.90 (1.16 to 5.98)	0.009	3.04 (2.60 to 3.54)	<0.001
Diabetes mellitus under 30 (250)	2.13 (1.33 to 3.26)	0.001	3.22 (1.79 to 5.54)	<0.001	2.58 (2.22 to 2.98)	<0.001
Goodpasture's syndrome (446.2)	2.29 (0.28 to 8.28)	0.50	6.89 (1.87 to 17.7)	<0.001	2.78 (1.78 to 4.14)	<0.001
Hashimoto's thyroiditis (245)	0.89 (0.38 to 1.75)	0.87	0.91 (0.19 to 2.67)	0.91	1.41 (1.15 to 1.72)	0.001
Idiopathic thrombocytopenia purpura (287.3)	2.04 (0.93 to 3.88)	0.05	1.58 (0.97 to 2.42)	0.05	2.09 (1.90 to 2.29)	<0.001
Multiple sclerosis (340)	2.24 (1.83 to 2.72)	<0.001	2.52 (2.00 to 3.13)	<0.001	2.14 (2.03 to 2.26)	<0.001
Myasthenia gravis (358)	2.04 (1.09 to 3.49)	0.02	2.34 (1.24 to 4.01)	0.003	2.26 (2.00 to 2.54)	<0.001
Myxoedema (244)	1.47 (1.27 to 1.70)	<0.001	1.36 (1.22 to 1.52)	<0.001	1.30 (1.27 to 1.33)	<0.001
Pemphigus, pemphigoid (694.4,694.5)	2.69 (1.90 to 3.70)	<0.001	3.28 (2.07 to 4.94)	< 0.001	2.22 (2.00 to 2.45)	<0.001
Pernicious anaemia (281)	1.20 (0.99 to 1.44)	0.06	1.44 (0.97 to 2.07)	0.06	1.36 (1.27 to 1.45)	<0.001
Polyarteritis nodosa (446)	2.88 (1.70 to 4.55)	<0.001	4.36 (0.90 to 12.8)	0.03	3.53 (2.76 to 4.44)	<0.001
Primary biliary cirrhosis (571.6)	1.29 (0.52 to 2.67)	0.64	2.76 (1.37 to 4.95)	0.001	1.49 (1.26 to 1.74)	<0.001
Psoriasis (696.0,696.1,696.8,696.9)	1.62 (1.32 to 1.98)	<0.001	1.65 (1.23 to 2.17)	<0.001	1.66 (1.57 to 1.75)	<0.001
Rheumatoid arthritis (714)	1.45 (1.31 to 1.60)	<0.001	1.57 (1.37 to 1.79)	<0.001	1.75 (1.70 to 1.80)	<0.001
Scleroderma (710.1)	2.16 (0.99 to 4.10)	0.03	1.53 (0.70 to 2.91)	0.29	1.97 (1.73 to 2.23)	<0.001
Sjogren's syndrome (710.2)	2.85 (1.37 to 5.25)	0.001	1.91 (1.02 to 3.28)	0.03	2.02 (1.80 to 2.26)	<0.001
Systemic lupus erythematosus (710.0)	3.61 (2.36 to 5.31)	<0.001	4.60 (3.19 to 6.43)	<0.001	3.71 (3.43 to 4.02)	<0.001
Thyrotoxicosis (242)	1.14 (0.95 to 1.36)	0.14	1.56 (1.23 to 1.95)	<0.001	1.34 (1.27 to 1.42)	<0.001

^1 ^Adjusted for sex, age in five-year bands, time-period in single calendar years and district of residence in the ORLS datasets; and for sex, age in five-year bands, time-period in single calendar years, region of residence and deprivation score associated with patients' area of residence, in quintiles, in the England dataset. The rate ratios are calculated as the ratio of the observed/expected number in the cohort for each immune-mediated disease (see Appendix) to the observed/expected numbers in the reference cohort as calculated in the analysis undertaken for each of the individual disease (data not shown).

^2 ^Conditions used in reference cohort, with Office of Population, Censuses and Surveys (OPCS) code edition 3 for operations and ICD9 code for diagnosis) (with equivalent codes used for other coding editions): appendicectomy (OPCS3 441), squint (ICD9 378), otitis externa, otitis media (380 to 382), haemorrhoids (455), deflected septum, nasal polyp (470 to 471), impacted tooth and other disorders of teeth (520 to 521), inguinal hernia (550)), ingrown toenail and other diseases of nail (703), sebaceous cyst (706.2), internal derangement of knee (717), bunion (727.1), dislocations, sprains and strains (830 to 839, 840 to 848), superficial injury and contusion (910 to 919, 920 to 924).

^3 ^ICD 9 codes for each immune-mediated disease (equivalent codes were used for cases coded in ICD Revisions 7, 8 and 10.)

People were included in the rheumatoid arthritis or reference cohort if they did not have an admission for VTE either before or at the same time as the admission for rheumatoid arthritis or the reference condition. We then searched the database for any subsequent NHS hospital care for, or death from, VTE in these cohorts. We considered that rates of VTE in the reference cohort would approximate those in the general population of the region while allowing for migration in and out of it (data on migration of individuals were not available).

### Statistical methods

We calculated rates of VTE based on cohort analysis. For each broad age group of people with rheumatoid arthritis, and for VTE, we took "date of entry" into each cohort as the date of first admission for rheumatoid arthritis, or reference condition, and "date of exit" as the date of first record of VTE, death, or the end of the data file (31 December 1998 for ORLS1; 31 March 2008 for ORLS2 and English HES), whichever was the earliest. In the ORLS cohorts, in comparing the rheumatoid arthritis cohort with the reference cohort, we first calculated rates for VTE, stratified and then standardised by age (in five-year age groups), sex, calendar year of first recorded admission, and district of residence, to ensure that the results of group comparisons were equivalent in these respects. We used a similar approach to standardisation in the England dataset, stratifying by age (in five-year age groups), sex, calendar year of first recorded admission, region of residence, and quintile of patients' Index of Deprivation score (as a measure of socio-economic status). We used the indirect method of standardisation, using the combined rheumatoid arthritis and reference cohorts as the standard population. We applied the stratum-specific rates in the combined rheumatoid arthritis and reference cohorts to the number of people in each stratum in the rheumatoid arthritis cohort, separately, and then to those in the reference cohort. We calculated the ratio of the standardised rate of occurrence of VTE in the exposure cohorts relative to that in the reference cohort. The confidence interval for the rate ratio and χ^2 ^statistics for its significance were calculated as described elsewhere [13]. Calculated in this way, the rate ratio provides a measure of relative risk of VTE in the rheumatoid arthritis cohort, compared with the reference cohort. The fact that there is unmeasured migration in the populations covered by the study, and the use of an internal reference cohort for comparison with the VTE rates in the rheumatoid arthritis cohort (and others), preclude meaningful calculation of absolute risks.

We analysed the occurrence of an admission for VTE within 90 days of the admission for each immune-related disease, and at 91 days and more, to help establish whether any elevated risk of VTE was confined to the short-term after the episode of hospitalisation or was more prolonged.

In the analysis of diabetes mellitus, we used hospital admission for diabetes mellitus when aged under 30 as a proxy for type 1 diabetes, as the type of diabetes is not well recorded in routine hospital statistics. We analysed the data for males and females separately, as well as together, to ascertain whether or not there were differences between them in risk of VTE.

## Results

Table 1 shows the number of people in the study who were admitted to the hospital with each of the selected immune-mediated diseases; it also shows the percentage of these who were female. The number of people in each of the three corresponding comparison cohorts were: 313,716 (47% female) for ORLS1, 187,609 (46% female) for ORLS2, and 3,707,315 (41% female) for England.

There were elevated risks of VTE after hospital admission for the following individual immune-mediated diseases, in all three of the populations studied: autoimmune haemolytic anaemia, chronic active hepatitis, dermatomyositis/polymyositis, type 1 diabetes mellitus, multiple sclerosis (MS), myasthenia gravis, myxoedema, pemphigus/pemphigoid, polyarteritis nodosa, psoriasis, rheumatoid arthritis, Sjogren's syndrome, and SLE (Table 2 and Appendix).

In the much larger population of England, we also found elevated risks for VTE in people admitted with Addison's disease, ankylosing spondylitis, coeliac disease, Goodpasture's syndrome, Hashimoto's thyroiditis, idiopathic thrombocytopenia purpura, pernicious anaemia, primary biliary cirrhosis, scleroderma and thyrotoxicosis (Table 2).

High levels of risk, substantially higher than the risks associated with some of the other diseases, were found for SLE and polyarteritis nodosa. The rate ratios for SLE were 3.61 (2.36 to 5.31) in the ORLS1 population, 4.60 (3.19 to 6.43) in ORLS2 and 3.71 (3.43 to 4.02) in the English national dataset. Those for polyarteritis nodosa were, respectively, 2.88 (1.70 to 4.55), 4.36 (not quite significant with CIs of 0.90 to 12.8) and 3.53 (2.76 to 4.44). Consistently high levels of risk were also found for chronic active hepatitis, dermatomyositis and polymyositis, diabetes mellitus, MS and Sjogren's syndrome.

### Males and females

On the whole, the rate ratios for males and females were similar although some of the numbers in ORLS1 and ORLS2 became rather small when subdivided by sex. In the England dataset, there was a significant elevation of VTE risk in males with coeliac disease (RR 1.47; 1.26 to1.70), but not in females (1.09; 0.97 to 1.23). Similarly, the VTE rate ratio for males with Hashimoto's thyroiditis was significantly elevated (2.98; 1.86 to 4.51), but the rate ratio in females was not (1.23; 0.97 to 1.54). There were no other significant differences between males and females.

### Short (0 to 90 days) and long-term (91+ days) associations with VTE

We studied the occurrence of VTE in time intervals after admission. However, subdividing the results into 0 to 90 days and 91+ days since immune-mediated disease admission did reduce power. Only MS, myxoedema, psoriasis, rheumatoid arthritis and SLE had sufficient numbers for meaningful analysis. Table 3 shows that, in general, associations with increased VTE risk were found for these diseases at both short and long intervals.

**Table 3:** Occurrence of VTE within 90 days or more than 90 days after admission for immune-mediated disease.

Exposure, time between exposure and VTE (days)	ORLS1			ORLS2			England		
	Obs	Exp	RR^1 ^(95% CI)	Obs	Exp	RR^1 ^(95% CI)	Obs	Exp	RR^1 ^(95% CI)
MS 0 to 90	10	8.1	1.25 (0.59 to 2.31)	17	6.7	2.64 (1.51 to 4.33)	212	112	1.95 (1.69 to 2.24)
91+	96	43.7	2.23 (1.80 to 2.74)	71	30.1	2.45 (1.90 to 3.13)	1297	619	2.17 (2.05 to 2.30)
Myxoedema 0 to 90	38	39.8	0.95 (0.66 to 1.32)	126	95.9	1.48 (1.18 to 1.85)	2617	2058	1.48 (1.41 to 1.56)
91+	160	107	1.52 (1.29 to 1.79)	305	245	1.32 (1.16 to 1.51)	8487	7237	1.26 (1.22 to 1.29)
Psoriasis 0 to 90	12	11.6	1.04 (0.53 to 1.83)	15	6.5	2.41 (1.33 to 4.06)	226	134	1.73 (1.51 to 1.98)
91+	87	54.2	1.62 (1.29 to 2.00)	38	26.5	1.45 (1.02 to 2.01)	1092	679	1.64 (1.54 to 1.75)
Rheumatoid arthritis: 0 to 90	52	61.7	0.83 (0.61 to 1.10)	50	37.0	1.43 (1.03 to 1.95)	1122	730	1.71 (1.60 to 1.83)
91+	395	279.8	1.46 (1.31 to 1.62)	231	156	1.60 (1.38 to 1.85)	5703	3626	1.75 (1.70 to 1.81)
SLE 0 to 90	5	1.4	3.64 (1.18 to 8.56)	6	1.8	3.41 (1.24 to 7.75)	111	27.4	4.15 (3.40 to 5.01)
91+	21	6.5	3.25 (2.01 to 4.98)	29	5.9	5.03 (3.35 to 7.27)	525	148	3.61 (3.31 to 3.94)

^1 ^Adjusted for sex, age in five-year bands, time-period in single calendar years and district of residence in the ORLS datasets; and for sex, age in five-year bands, time-period in single calendar years, region of residence and deprivation score associated with patients' area of residence, in quintiles, in the England dataset.

### Absolute risk

Although we could not calculate meaningful absolute risks for the ORLS1 and ORLS2 cohorts, because of migration into and out of the Oxford region, approximate absolute risks can be calculated in the English national data. For example, there were 81,950 people in the MS cohort of whom 1,509 had an admission for VTE (1.8%) during the 10-year period covered by the study. As an approximation, in people with MS there was one case of VTE per 264 person-years at risk. Similar calculations show that 2.7% of people with SLE had an admission for VTE in the period covered by the study, and that the event rate was one case of VTE per 169 person-years at risk. These calculations assume that migration was small and that its effect was unimportant.

## Discussion

The ORLS1 (1963 to 1998) and ORLS2 (1999 to 2008) cover broadly the same population but at different times. The English linked dataset is completely independent of ORLS1 and it covers a far larger population in the same timeframe as ORLS2. The results of the analyses in the three datasets corroborate each other. Previous studies have found elevated risks of VTE in people with rheumatoid arthritis [6], type 1 diabetes mellitus [7], SLE[8] and inflammatory bowel disease [5]. We have not reported on inflammatory bowel diseases here as they are included in a different epidemiological study that we hope to publish separately. These previous findings, combined with our own, suggest that there may be a general association between immune-mediated diseases and the risk of subsequent VTE. This risk is not solely associated with the short term after hospital admission: for the diseases with large enough numbers to study, it was sustained over time.

The increased risks of VTE may have different underlying causes in each disease. The elevated risks may be a reflection of patients with more extreme cases of the immune-mediated diseases, in that the populations in our study are those admitted to hospital. Immobility [14], effects of treatment (corticosteroids promote haemostasis) [15] or a true effect of inflammation on coagulation [16] could all be implicated in the associations shown.

In 1856, Virchow proposed three precipitants for venous thrombosis: venous stasis, increased coagulability of the blood, and damage to the vessel wall [17]. Inflammation is a key determinant of endothelial function in both arteries and veins and results in changes in expression of selectins and cellular adhesion molecules [16]. Studies have shown that patients with VTE were more likely to have elevated plasma IL-8, IL-6, MCP-1 and TNF-α levels [18], that inflammation influences clotting factor levels [19], that an inflammatory gene is associated with VTE [20] and acute inflammation does contribute to VTE [21]. Taken together, inflammation is likely to contribute to some extent to the initiation of venous thrombus formation.

### Methodological issues: datasets, population, and multiple comparisons

Strengths of the datasets include their size, with large numbers of fairly uncommon diseases. The ORLS1 data provide long duration of follow-up; the English data provide a much larger and more recent population but with shorter follow-up. The risk of VTE was therefore studied for a large number of immune-mediated diseases, all within a single population and using the same methodology. Accordingly, levels of risk associated with different immune-mediated diseases can be directly compared within the same study populations.

Our study should be regarded as exploratory rather than definitive. We studied a wide range of immune-mediated diseases with differing aetiologies. We did so because, as an exploratory study, with very large linked datasets in which many diseases can be studied, we saw no reason to be restrictive in our selection of diseases.

The datasets have limitations. The cohorts are based on prevalent cases, the first recorded hospital admission or episode of day case care for each person with each condition, rather than being cohorts with follow-up from the date of first diagnosis. Data are not recorded on patients who move out of the area covered by data collection or who are treated in hospitals outside the area (mainly affecting ORLS). The datasets are limited to people who were admitted to hospital, or who received day case specialist care. This would not capture all people with each immune-mediated disease, although it should identify the great majority with subsequent diagnosed VTE. These factors are part of our reasoning for including a comparison cohort of patients admitted to hospital, or in receipt of day case care, from the same database and for 'matching', through stratified analysis, for area of treatment and for year of first recorded diagnosis as well as for age and sex. The two Oxford datasets are not linked to each other due to changes in the data items available for linkage between 1998 and 1999. Consequently, it is likely that some people have been recorded as having a 'first admission' for each immune-mediated disease in each of the time periods studied.

We lack clinical and laboratory data. We lack treatment data for the immune-mediated diseases; and elements of their treatment could themselves influence VTE risk. There is very limited information on potential confounding factors such as socioeconomic status, and none on smoking or ethnicity. As we comment above, our results should be regarded as speculative rather than definitive: they represent results from what can be done using very large-scale, routinely collected administrative data. They need further work, in different study designs, to confirm or refute the findings, although epidemiological studies involving direct patient contact may be quite formidable logistical undertakings on the scale required.

Our rate ratio for VTE in people admitted to hospital with rheumatoid arthritis in the English dataset was 1.75, which is comparable with a previous study that reported a relative risk of 1.99 [6]. Our rate ratio for VTE in people aged under 30 with diabetes was 2.58 in the English dataset, which compares with a reported figure of 1.73 in hospitalised patients with diabetes aged 20 to 29 in the USA [7]. Although the literature is sparse on VTE in people with immune-mediated diseases, our findings seem broadly comparable with findings of others.

We used age at admission under 30 as a proxy for type 1 diabetes mellitus, as the type of diabetes is not routinely recorded on hospital admission records. Although this will mostly consist of people with type 1 diabetes, there may be a few people with type 2 diabetes in the cohort as well.

We studied a large number of associations between diseases. The effect of making multiple comparisons needs to be considered. For this reason, we have given exact *P-*values, as well as confidence intervals, so that the reader can judge the degree of significance of each immune-mediated disease and subsequent VTE. It is possible that some of the associations that are significant at a level of *P *< 0.05 or *P *< 0.01 may result from making multiple comparisons and the play of chance. This may particularly be so where there is no prior hypothesis to support the finding. On the other hand, even in a study with the number of comparisons that we have made, findings where the significance level is <0.001 or less, are unlikely to be attributable to chance alone. There were differences in levels of risk between the diseases studied: for example, the rate ratios for SLE were significantly and substantially higher than those for myxoedema. Even if the fairly low levels of elevation of risk, such as those associated with myxoedema and thyrotoxicosis, are considered unimportant, the high levels of risks associated with SLE, polyarteritis nodosa and diabetes mellitus are striking. The large numbers of findings with highly significant results in the England cohort needs comment. They no doubt in part reflect the very large numbers of patients in the cohort, such that many differences are significant, even if fairly small (for example, coeliac disease, thyroiditis, myxoedema, thyrotoxicosis), as a result of high statistical power.

## Conclusion

This is an exploratory study into the risk of VTE in people admitted to hospital with a range of immune-mediated diseases. Further studies are needed, of individual immune-mediated diseases, in greater depth, to confirm or refute our findings. Given that a large proportion of hospitalised patients are at risk for VTE, and that there is, generally, a low rate of appropriate prophylaxis, our data suggest that patients with selected immune-mediated disease may need to be considered for thromboprophylaxis. This may be particularly warranted in people with diseases at relatively high risk of VTE, such as SLE and polyarteritis nodosa. However, the risk of VTE for any immune-mediated disease we studied is at least an order of magnitude lower than the risk of VTE after surgery[22]. Further investigation is required, prospectively, to determine the thromboprophylaxis status of patients with these immune-mediated diseases who experience VTE. If a substantial proportion of such patients are found not to have been receiving thromboprophylaxis, current recommendations on thromboprophylaxis may need to be re-examined, as the disorders we studied here were not included as risk factors in the recent NICE guidelines on reducing the risk of VTE [23]. Prospective studies are needed to determine the predictive value of inflammatory markers for VTE, but this may also aid in the identification of individuals who strongly warrant preventative therapy.

## Abbreviations

CI: confidence interval; DVT: deep vein thrombosis; HES: Hospital Episode Statistics; ICD: International Classification of Diseases; IL: interleukin; MCP: monocyte chemoattractant protein; MS: multiple sclerosis; NHS: National Health Service; NICE: National Institute for Health and Clinical Excellence; P = probability; PE: pulmonary embolism; SLE: systemic lupus erythematosus; TNF: tumour necrosis factor; VTE: venous thromboembolism

## Competing interests

The authors declare that they have no competing interests.

## Authors' contributions

MJG is the guarantor of and designed the study. SVR, MJG and CJW conceived the study. CJW led the analysis. SVR, AEH, DY contributed to the analysis and interpretation of the data. SVR and CJW wrote the first draft and all contributed to subsequent drafts and the final paper.

## Appendix

Observed (O) and Expected (E) numbers of people with an admission for DVT or PE in people with selected immune-mediated diseases (see Table 4).

**Table 4:** Observed (O) and Expected (E) numbers of people with an admission for DVT or PE

	Dataset (years)					
	
Immune-mediated disease (ICD code^3^)	ORLS1 (1963 to 1998)		ORLS2 (1999 to 2008)		England (1999 to 2008)	
	**O**	**E**	**O**	**E**	**O**	**E**
Addison's disease (255.4)	13	8.7	12	4.1	205	95.9
Ankylosing spondylitis (720)	18	13.4	10	8.5	373	194.7
Autoimmune haemolytic anaemia (283)	16	5.7	17	4.7	353	93.4
Chronic active hepatitis (571.4)	15	6.7	6	1.5	93	45.7
Coeliac disease (579.0)	27	20.0	24	17.8	474	392.0
Dermatomyositis, polymyositis (710.3,710.4)	11	5.6	7	2.4	167	55.2
Diabetes mellitus under 30 (250)	23	11.2	18	6.7	224	96.9
Goodpasture's syndrome (466.2)	2	0.9	4	0.6	24	8.6
Hashimoto's thyroiditis (245)	8	9.0	3	3.3	100	70.9
Idiopathic thrombocytopenia purpura (287.3)	9	4.4	21	13.4	465	225.0
Multiple sclerosis (340)	106	48.2	88	36.6	1509	728.8
Myasthenia gravis (358)	13	6.4	13	5.6	282	125.9
Myxoedema (244)	202	139.8	431	342.2	11104	9284.7
Pemphigus, pemphigoid (694.4,694.5)	38	14.2	23	7.1	375	170.8
Pernicious anaemia (281)	120	100.5	30	21.0	1012	754.2
Polyarteritis nodosa (446)	18	6.3	3	0.7	73	20.7
Primary biliary cirrhosis (571.6)	7	5.4	11	4.0	150	101.2
Psoriasis (696.0,696.1,696.8,696.9)	99	61.7	53	32.8	1318	812.5
Rheumatoid arthritis (714)	450	322.1	281	192.9	6825	4352.5
Scleroderma (710.1)	9	4.2	9	5.9	244	124.8
Sjogren's syndrome (710.2)	10	3.5	13	6.8	305	152.2
Systemic lupus erythematosus (710.0)	26	7.2	35	7.8	636	174.8
Thyrotoxicosis (242)	134	117.8	83	54.7	1429	1081.7

## Pre-publication history

The pre-publication history for this paper can be accessed here:

http://www.biomedcentral.com/1741-7015/9/1/prepub
